# Detecting Colorectal Neoplasia Using Specific Fecal Fluorogenic Protease-Sensitive Substrates: A Pilot Study

**DOI:** 10.1021/acs.analchem.4c04586

**Published:** 2024-12-12

**Authors:** Roza C. M. Opperman, Sofie Bosch, Kamran Nazmi, Floris J. Bikker, Henk S. Brand, Connie R. Jimenez, Tim G. J. de Meij, Evelien Dekker, Nanne K. H. de Boer, Wendy E. Kaman

**Affiliations:** † Department of Gastroenterology and Hepatology, Amsterdam UMC, Vrije Universiteit Amsterdam, 1081 HV Amsterdam, The Netherlands; ‡ Amsterdam Gastroenterology Endocrinology Metabolism (AGEM) Research Institute, 1081 HV Amsterdam, The Netherlands; § Research Program, Cancer Center Amsterdam, 1081 HV Amsterdam, The Netherlands; ∥ Department of Oral Biochemistry, Academic Centre for Dentistry Amsterdam, University of Amsterdam and VU University Amsterdam, Gustav Mahlerlaan 3004, 1081 LA Amsterdam, The Netherlands; ⊥ Department of Medical Oncology, Amsterdam UMC, VU University Medical Center, 1081 HV Amsterdam, The Netherlands; # Department of Pediatric Gastroenterology, Emma Children’s Hospital, Amsterdam UMC, Vrije Universiteit Amsterdam, 1081 HV Amsterdam, The Netherlands; ¶ Department of Pediatric Gastroenterology, Emma Children’s Hospital, Amsterdam UMC, Academic Medical Centre, 1105 AZ Amsterdam, The Netherlands; ∇ Department of Gastroenterology and Hepatology, Amsterdam UMC, University of Amsterdam, 1081 HV Amsterdam, The Netherlands

## Abstract

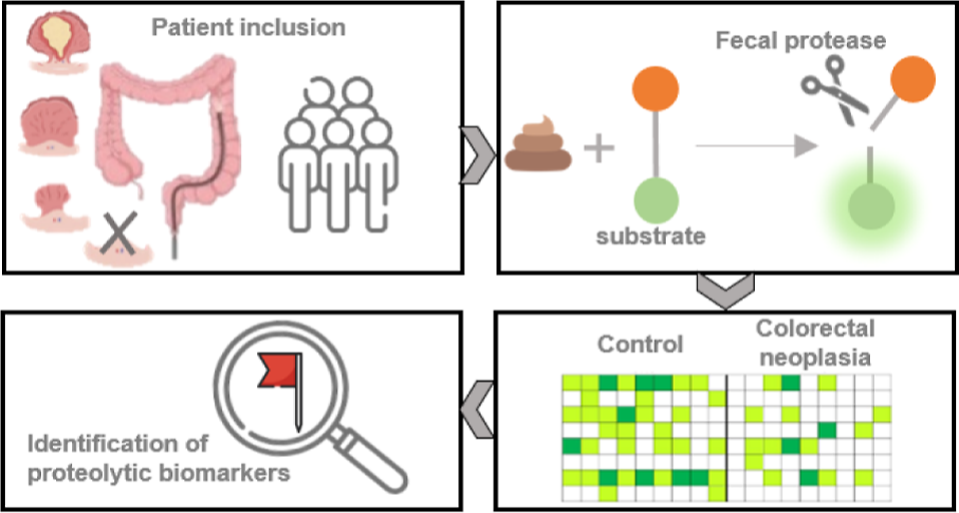

Background: identification and removal of advanced adenomas (AA) reduce colorectal cancer (CRC) incidence and potentially mortality. CRC screening often uses fecal immunochemical testing to select high-risk individuals for colonoscopy, despite its low sensitivity for AA and relatively high false-positivity rate. Previous studies have linked proteases to CRC development through their ability to facilitate angiogenesis and immunoregulation. This study aims to identify colorectal neoplasia-associated proteases and their substrates as a potential noninvasive screening test, introducing an innovative application of fecal protease profiling, which has previously been limited to tissue samples. Methods: eighteen fluorogenic substrates were designed based on literature. Proteolytic degradation of these substrates was measured in fecal samples of patients with CRC (*n* = 12), AA (*n* = 9), nonadvanced adenomas (*n* = 10), and controls (*n* = 14). Substrate degradation was correlated to a matched human proteome data set, and underlying proteases were identified based on their recognition patterns. Experiments with protease inhibitors and ZnCl_2_ were performed to further characterize the involved proteases. Results: in total, 7 of the 18 substrates tested showed a significantly decreased proteolytic degradation in feces from patients with any colorectal neoplasia compared to the control group. The l-aspartic acid–l-glutamic acid substrate (ED) showed significantly decreased degradation in AA and CRC patients. ED degradation significantly decreased with the addition of ZnCl_2_ and the cysteine protease inhibitor NEM. Conclusion: we successfully developed colorectal neoplasia-specific fluorogenic substrates, highlighting the ED substrate as a potential substrate for the detection of AA and CRC. Although the responsible proteases require further identification, our results suggest an association with calcium-dependent cysteine proteases.

## Introduction

Colorectal cancer (CRC) is one of the main causes of cancer-related deaths worldwide.^1^ Tumors typically arise from benign polyps via the adenoma–carcinoma sequence (85%) and, to a lesser extent, via the serrated pathway (15%).^2^ As the 5-year survival rate decreases rapidly with disease stage at diagnosis, early detection and endoscopic removal of (pre)malignant colorectal neoplasia reduces the CRC incidence and may lower CRC-related deaths.^3^ As a result, many countries have implemented population-based screening programs. Currently, fecal immunochemical testing (FIT) is one of the most widely used screening tests in European countries and showed to reduce CRC incidence.^4,5^ However, within the Dutch screening program, approximately 70% of FIT-positive patients do not exhibit advanced colorectal neoplasia at subsequent colonoscopy.^6^ This leads to many unnecessary, invasive, and burdensome colonoscopies.^5,7^ Furthermore, although strongly dependent on the cutoff value, FIT is not sufficiently sensitive for the detection of advanced adenomas (AA), illustrated by 50–82% missed cases.^8^ This underlines the need for complementary and accurate noninvasive tests.

In several studies, the production and release of numerous proteases in the tumor microenvironment has been observed during development, progression, and metastasis of cancer.^9^ Proteases are involved in the regulation of a broad variety of cellular processes including cell signaling, tissue remodeling, and immune function.^9,10^ A number of studies showed that the presence of specific proteases and CRC development are correlated, i.e., cysteine and serine proteases are upregulated in CRC tissue.^11,12^ In addition, matrix metalloproteinases have been detected in CRC tissue and are mainly involved in the degradation of extracellular matrix components, enabling tumor invasion and metastasis.^13,14^

The high abundance of proteases in both gastrointestinal tissue and the microbiome makes stool an ideal source for noninvasive testing. In addition, proteolytic profiling of protease substrates is a straightforward technique, underlying its suitability to develop an accessible and easy to use diagnostic test. In the current study, we aim to identify specific fecal proteolytic profiles using fluorogenic substrates as a potential diagnostic test for colorectal neoplasia detection. Furthermore, we aim to gain insight into the cleavage characteristics of the underlying proteases.

## Materials and Methods

### Study Population and Fecal Samples

In the current study, data and fecal samples were used from a case–control study that was reviewed and approved by the Medical Ethical Review Committee (METC) of the Amsterdam UMC (2014.404).^15^ All participants provided written informed consent prior to their participation in the study. In short, between 2016 and 2019, patients scheduled for colonoscopy at three Dutch hospitals, regardless of indication, were requested to participate in the study. One week prior to colonoscopy, a fecal sample was collected at home, which was stored at −20 °C within 1 h after sampling, until it was transported to the hospital under cooled conditions. Furthermore, participants completed a questionnaire including patient demographics. Based on observations at colonoscopy and on histopathological results, patients were divided into four different subgroups: (a) CRC: patients with histopathologically proven adenocarcinoma of the colon or rectum, (b) AA: adenomas ≥1 cm in diameter or with ≥25% villous histology or with high grade dysplasia, (c) nonadvanced adenomas (nAA): adenomas 0.5–0.9 cm, without villous histology and high grade dysplasia, and (d) controls (C): patients with no bowel abnormalities observed during colonoscopy (excluding hemorrhoids and/or diverticula). Patients with colorectal cancer were treatment naïve at the time of sampling. Exclusion criteria were prior diagnosis of an underlying gastrointestinal disease (e.g., inflammatory bowel disease and celiac disease), incomplete endoscopic assessment, or insufficient fecal mass to perform analysis.

### Substrate Design and Proteolytic Profiling of Fecal Samples

Substrates were designed based on the literature describing significantly elevated levels of certain amino acids in fecal samples from patients with adenomas and CRC, indicating the presence of proteases capable of cleaving sequences containing these amino acids. These substrates consist of l-amino acids, d-amino acids, or a combination, which are two different optical isomers: levorotary (l) and dextrorotary (d). All substrates were synthesized as described elsewhere; the substrates were N-terminally flanked with an aminohexanoic acid (Ahx)-linked fluorescein isothiocyanate (FITC) and C-terminally flanked with a lysine-coupled quencher [Dabcyl (Dbc)].^16^ Most substrates are part of a previously published library^17^ (Table 2.). These substrates were purchased from PepScan Presto (Lelystad, The Netherlands) with a purity >90%, and the structural characterization of the substrates was confirmed using mass spectrometry. The purity of the newly synthesized substrates was confirmed with high-performance liquid chromatography (HPLC) using a combination of LC-NETII/ADC, PU-1580, LG-1580-02, and UV-1575 (JASCO Benelux B.V., de Meern, The Netherlands). The mass of the newly synthesized substrates were confirmed using a Microflex LRF MALDI-TOF MS (Bruker, Billerica, MA, USA) (Table 2 and Figures S1 and S2). Fecal proteolytic activity was analyzed using a fluorescence resonance energy transfer (FRET) assay, as described previously.^18^ In short, 700 mg feces was dissolved in 7 mL sample buffer [20 mM HEPES with 0.05% Tween 20 (pH 8.2)] and mixed for 20 s to homogenize the samples. From this solution, 49 μL was incubated with 1 μL of substrate, and the increase in fluorescence was monitored using a fluorescence microplate reader (FLUOstar Galaxy, BMG Laboratories, Offenburg, Germany). Substrate cleavage was calculated by the fluorescence (*F*) emitted per minute for each sample. The slope was determined over two time intervals: 0–20 and 0–60 min. A result was considered positive if the proteolytic activity reached or exceeded 5.0 *F*/min. The highest calculated slope was used for the statistical analysis.

### Protease Identification and Cleavage Characteristics

In order to identify the proteases, responsible for the observed proteolytic activity, significant fecal proteolytic profiles were correlated with human proteome data, which were available for all fecal samples.^15^ Stool proteomics was performed with liquid chromatography–tandem mass spectrometry (LC–MS/MS), as described previously.^15^ The cleavage preferences of the correlated proteases were then searched in the MEROPS and Uniprot databases to determine their ability to cleave the substrate.^19,20^ To study the characteristics of the proteases responsible for the observed proteolytic activity, the effect of various chemical compounds was individually tested, including 250 μM zinc chloride (ZnCl_2_), 5 mM benzamidine (BAM), 0.4 mM Pefabloc, and 12.5 mM *N*-ethylmaleimide (NEM), all in purified water and 0.2 mM ethylenediaminetetraacetic acid (EDTA) in HEPES buffer. These compounds were selected for the following reasons: ZnCl_2_ binds at the calcium ion binding site, thereby deactivating the activity of calcium-dependent proteases; BAM acts as a trypsin-like serine protease inhibitor; Pefabloc serves as a serine protease inhibitor; NEM inhibits cysteine proteases; and EDTA is a metalloprotease inhibitor. All chemicals were purchased from Sigma-Aldrich (Saint Louis, Missouri, United States).

### Statistical Analyses

Data visualization was conducted with R (version 4.2.1), using the packages ggplot 2 and corrplot. All statistical analyses were performed using RStudio (version 4.2.1). Differences in median were analyzed using the Kruskal–Wallis test, using pairwise Wilcoxon rank-sum test as post hoc analysis. Categorical variables were analyzed using the Fisher’s exact test. Differences in fecal proteolytic profiles were analyzed using the Mann–Whitney *U*-test. Spearman correlation was used to analyze correlations between substrates with significantly different proteolytic activity and the human proteome. Differences in proteolytic activity before and after the addition of protease inhibitor were analyzed using the Wilcoxon signed-rank test. A *p*-value ≤0.05 was considered statistically significant.

## Results

### Patient Demographics

A total of 45 fecal samples, originating from 45 participants (12 with CRC, 9 with AA, 10 with nAA, and 14 controls), were available for protease analysis. There were no significant differences between the study groups regarding age, gender, BMI, smoking status, and antibiotic use. The groups of patients did not differ in the location of the largest lesion. Detailed participant characteristics are depicted in Table 1.

**Table 1:** Demographics of Study Participants^a^

	CRC (12)	AA (9)	nAA (10)	control (14)
age (median [IQR])	67 [61–70]	71 [70–73]	73 [63–75]	68 [62–74]
gender (male *N* [%])	6 [50]	8 [88.89]	8 [80]	10 [71.43]
BMI (median [IQR])^b^	25.6 [23.8–28.3]	26.2 [23–27.5]	26 [23.7–27.8]	25.8 [23.5–28.3]
smoking status (*N* [%])				
*never smoked*	3 [25]	1 [11.1]	2 [20]	4 [28.6]
*stopped smoking*^c^	8 [66.7]	7 [77.8]	6 [60]	8 [57.1]
*actively smoking*	1 [8.3]	1 [8.3]	2 [20]	2 [14.3]
antibiotic use <3 months (*N* [%])	1 [8.3]	1 [11.1]	1 [10]	4 [28.5]
endoscopy indication (*N* [%])				
*abdominal pain*	2 [16.7]	0 [0]	0 [0]	5 [35.7]
*surveillance after* polypectomy/CRC	0 [0]	1 [11.1]	3 [30]	2 [14.3]
*positive FIT*	4 [33.3]	2 [22.2]	2 [20]	3 [21.4]
*rectal blood loss*	3 [25]	3 [33.3]	0 [0]	0 [0]
*positive family history for CRC*	0 [0]	1 [11.1]	1 [10]	1 [7.1]
*change in defaecation pattern*	1 [8.3]	1 [11.1]	3 [30]	1 [7.1]
*other*^d^	2 [16.7]	1 [11.1]	1 [10]	2 [14.3]
location largest lesion (*N* [%])^e^				
*proximal (cecum–splenic flexure)*	2 [18.2]	2 [22.2]	5 [50]	NA
*distal (descending colon–rectum)*	8 [72.7]	6 [66.7]	5 [50]	NA
adenoma size, cm (median [IQR])^f^	NA	1.20 [1.10–1.90]	0.75 [0.53–0.80]	NA
AA characteristics (*N* [%])				
*high grade dysplasia*	NA	0 [0]	NA	NA
*>25% villous histology*	NA	2 [22.2]	NA	NA
*>1 cm*	NA	8 [88.9]	NA	NA
nAA characteristics (*N* [%])				
*no dysplasia*	NA	NA	0 [0]	NA
*low grade dysplasia*	NA	NA	10 [100]	NA
number of adenomas detected (median [IQR])	1 [1–4]	2 [2–4]	2 [1–2]	NA

a The median adenoma size was significantly higher in the AA group vs nAA group (*p* = 0.01).

b Information on BMI was missing for one participant of the AA and nAA groups.

c All patients quit smoking more than 10 years ago, except for one participant in the CRC and AA groups who recently quit (<1 year ago).

d Other is defined as anemia (one control participant), recurrent urinary tract infection (one control participant), coincidental radiologic findings (one CRC participant), and unclear (one participant in the CRC, AA, and nAA groups).

e Information on largest localization abnormality was missing for one participant in the CRC and AA groups.

f Carcinoma size was not included due to missing values of stenosing carcinomas. Abbreviations: AA = advanced adenoma, BMI = body mass index, CRC = colorectal cancer, FIT = fecal immunochemical test, IQR = interquartile range, NA = not applicable, and nAA = nonadvanced adenoma.

### Substrate Design

In our previous study, we observed significantly increased concentrations of the amino acids aspartic acid, glutamic acid, proline, and serine in fecal samples from adenoma patients compared to controls.^21^ In addition, the concentrations of threonine and valine decreased significantly following adenoma removal toward levels similar to controls, suggesting their adenoma-specific origin.^21^ Furthermore, fecal concentration of histidine was found to be elevated in stool samples of CRC patients compared to those with adenomas.^15^ Other studies also indicated higher histidine levels in both feces and tissue of CRC patients compared to controls.^22,23^ Based on these findings, a panel of 18 substrates was designed (Table 2). Six substrates containing glutamic acid were elongated or paired with aspartic acid to facilitate endopeptidase identification.

**Table 2:** Overview Fluorogenic Substrates Design and Substrate Sequence^a^

substrate abbreviation	fluorogenic substrate structure
DD^◊^	FITC–Ahx–l-aspartic acid–l-aspartic acid–LysDbc
Dd^◊^	FITC–Ahx–l-aspartic acid–d-aspartic acid–LysDbc
EE^◊^	FITC–Ahx–l-glutamic acid–l-glutamic acid–LysDbc
Ed^◊^	FITC–Ahx–l-glutamic acid–d-aspartic acid–LysDbc
ED^‡^	FITC–Ahx–l-glutamic acid–l-aspartic acid–LysDbc
DE^‡^	FITC–Ahx–l-aspartic acid–l-glutamic acid–LysDbc
PP^◊^	FITC–Ahx–l-proline–l-proline–LysDbc
SS^◊^	FITC–Ahx–l-serine–l-serine–LysDbc
VV^◊^	FITC–Ahx–l-valine–l-valine −LysDbc
TT^◊^	FITC–Ahx–l-threonine–l-threonine–LysDbc
HH^◊^	FITC–Ahx–l-histidine–l-histidine–LysDbc
Hd^◊^	FITC–Ahx–l-histidine–d-aspartic acid–LysDbc
EEE^‡^	FITC–Ahx–l-glutamic acid–l-glutamic acid–l-glutamic acid–LysDbc
EEEE^‡^	FITC–Ahx–l-glutamic acid–l-glutamic acid–l-glutamic acid–l-glutamic acid–LysDbc
EEEEE^‡^	FITC–Ahx–l-glutamic acid–l-glutamic acid–l-glutamic acid–l-glutamic acid–l-glutamic acid–LysDbc
EDED^‡^	FITC–Ahx–l-glutamic acid–l-aspartic acid–l-glutamic acid–l-aspartic acid–LysDbc
DEDE^‡^	FITC–Ahx–l-aspartic acid–l-glutamic acid–l-aspartic acid–l-glutamic acid–LysDbc
EEKKEE^‡^	FITC–Ahx–l-glutamic acid–l-glutamic acid–l-lysine–l-lysine–l-glutamic acid–l-glutamic acid–LysDbc

a Substrates that are part of the existing library are highlighted with a rhombus (◊), and the newly synthesized substrates are highlighted with a diesis (‡). Abbreviations: Ahx = aminohexanoic acid, FITC = fluorescein isothiocyanate, and LysDbc = lysine–Dabcyl.

### Proteolytic Activity Profiling

**Figure 1:**
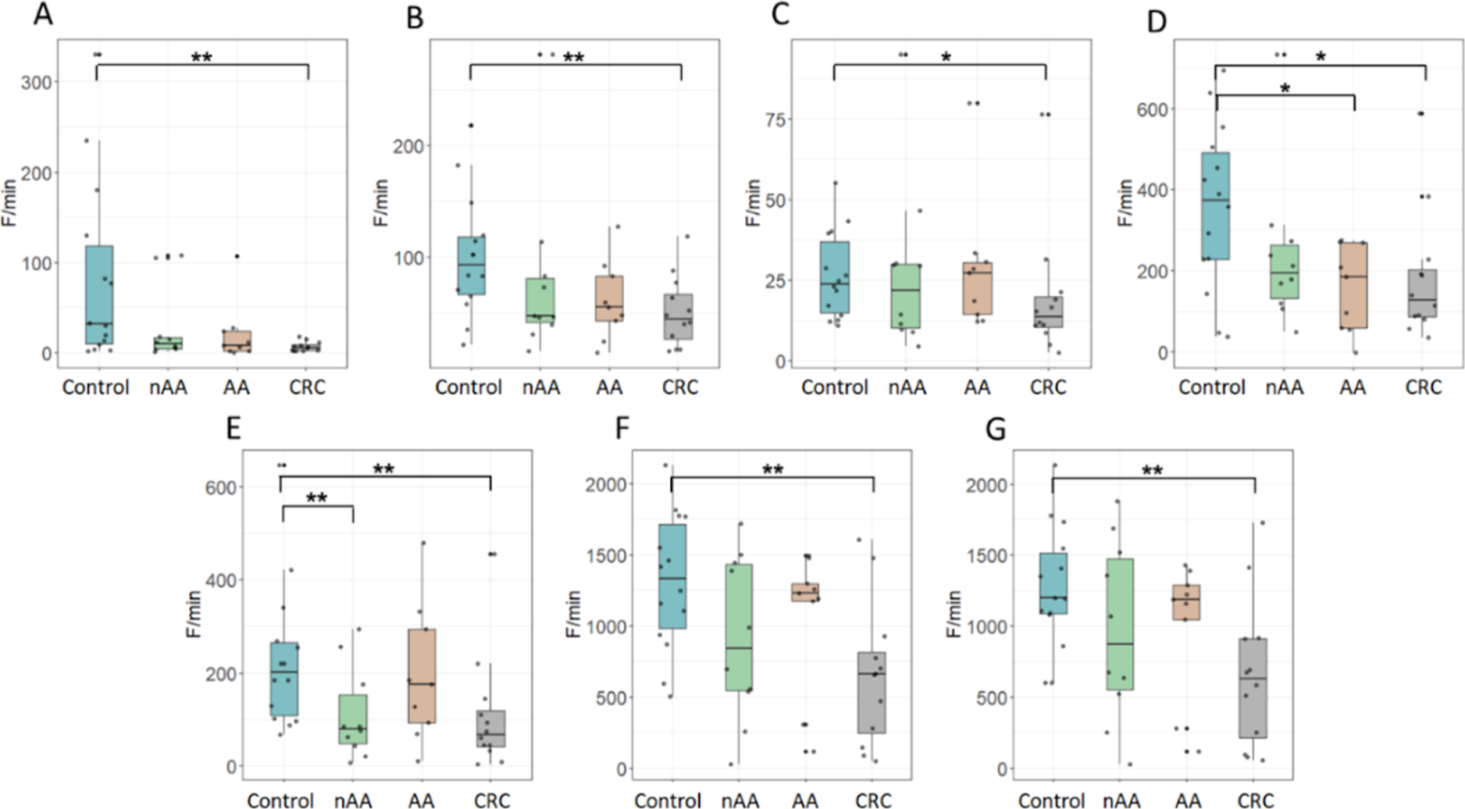
Fecal proteolytic degradation of substrates with a significant difference between study groups. Boxplots showing the fecal proteolytic activity for substrates HH (A), TT (B), DD (C), ED (D), EEE (E), EEEE (F), and EEEEE (G) in controls, patients with nAA, AA, and CRC. The points outside the whisker boundaries are considered outliers. Unpaired univariate analyses were performed using Mann–Whitney *U* test. Significance levels are depicted with an asterisk: *p < 0.05 and **p < 0.01. AA = advanced adenoma, CRC = colorectal carcinoma, and nAA = nonadvanced adenoma.

The proteolytic degradation of all 18 substrates was measured. A significantly reduced degradation of seven substrates was observed in fecal samples from CRC patients compared to control samples (Figure 1); HH (*p* = 0.004), TT (*p* = 0.009), DD (*p* = 0.046), ED (*p* = 0.027), EEE (*p* = 0.009), EEEE (*p* = 0.004), and EEEEE (*p* = 0.005). In addition, proteolytic activity was significantly decreased in the nAA group relative to controls for the EEE substrate (*p* = 0.02) and in the AA group compared to controls for the ED substrate (*p* = 0.02) (Figure 1D,E). For the other substrates, no significant difference in degradation was observed in the feces of the different research groups.

### Correlation between Proteolytic Activity and Human Proteome

**Figure 2:**
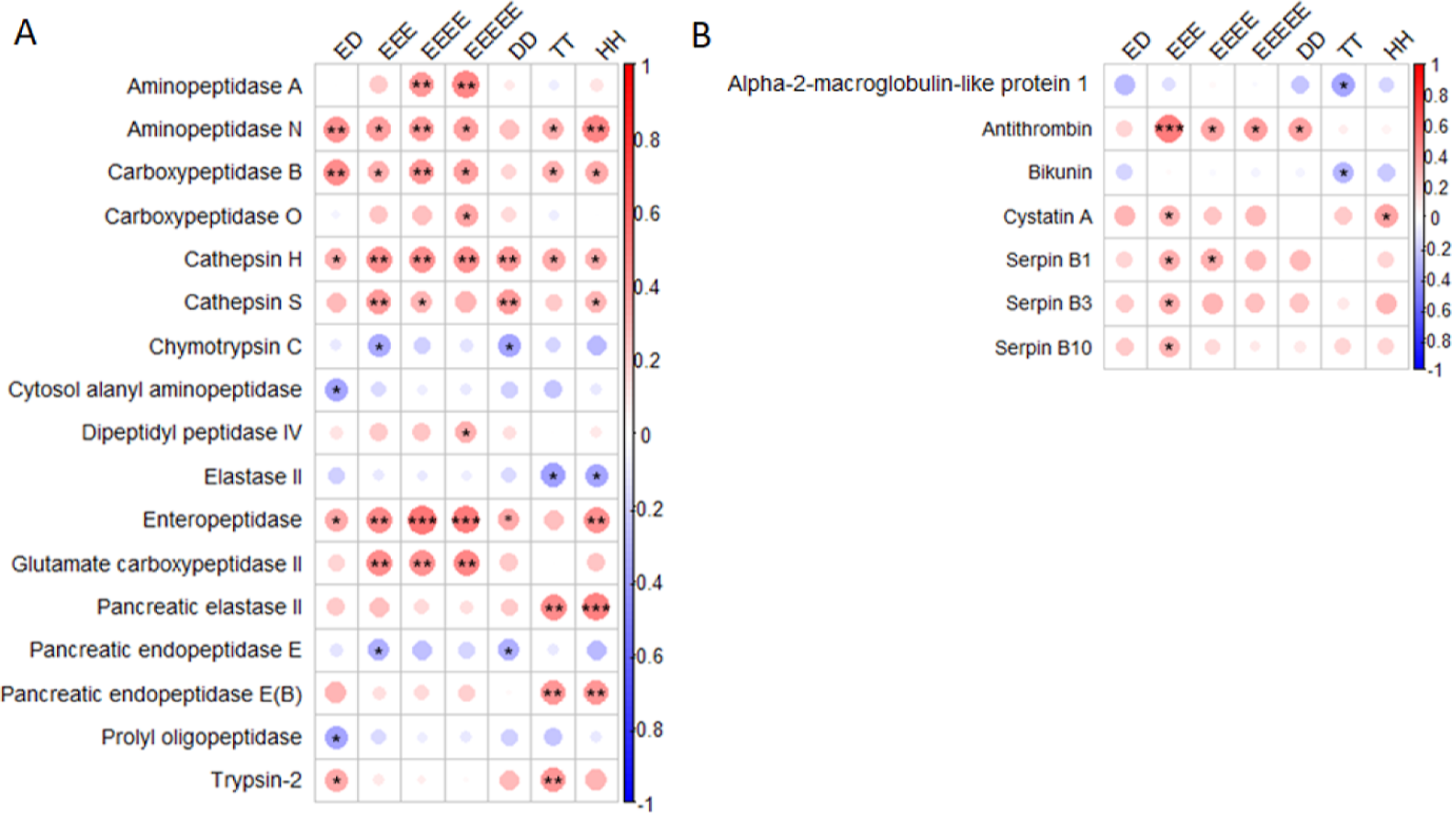
Correlation between fecal proteolytic activity of selected substrates and the fecal human proteome. Spearman’s correlation analyses for proteolytic activity of selected substrates with proteases (A) and natural protease inhibitors (B) as measured with LC–MS/MS for all study groups. Only significant correlations are depicted. As indicated by the bar on the right, negative correlations are in blue and positive correlations in red; the darker the color, the stronger the correlation. Significance levels are depicted with asterisks: *p < 0.05, **p < 0.01, and ***p < 0.001.

The degradation profiles of the seven substrates, which were significantly less degraded in fecal samples from patients with CRC, AA, or nAA, were correlated with the human proteome data from these samples, aiming to identify the proteases responsible for the observed alterations in proteolytic activity. The proteomics data set includes 469 human proteins in total.^15^ Spearman’s correlation analyses resulted in 261 significantly correlated proteins, comprising 18 proteases and 7 natural protease inhibitors. Significant positive correlations were found for aminopeptidase N, dipeptidyl peptidase 4, carboxypeptidase B, carboxypeptidase O, cathepsin H, cathepsin S, pancreatic elastase II, pancreatic endopeptidase E form B, enteropeptidase, glutamate carboxypeptidase II, aminopeptidase A, and trypsin 2 (Figure 2A and Table S1). Significant negative correlations were found for carboxypeptidase A2, chymotrypsin-C, pancreatic endopeptidase E, aminopeptidase A, elastase II, propyl oligopeptidase, and cytosol alanyl aminopeptidase. For all correlated proteases, substrate cleavage recognition preferences were derived from Uniprot and MEROPS database if available.^19,20^ Trypsin 2, an endopeptidase, exhibits nonspecific cleavage patterns and could therefore be matched to multiple substrates. Furthermore, based on the cleavage patterns, no other substrate—protease matches could be made. Moreover, significant positive correlations were found for the following natural protease inhibitors: antithrombin, cystatin A, serpin B1, B3, and B10. Negative correlations were found for α-2-macroglobulin-like protein 1 (A2ML1) and bikunin (Figure 2B and Table S1).

### Effect of Protease Inhibitors to the Proteolytic Activity of l-Aspartic Acid–l-Glutamic Acid Substrate

**Figure 3:**
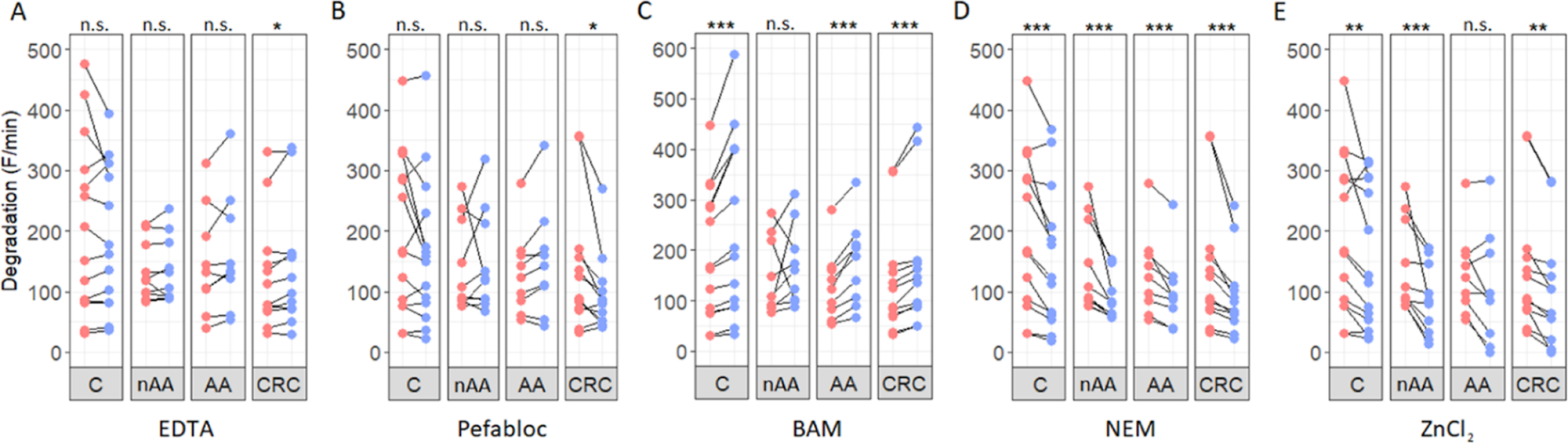
Effect of the addition of protease inhibitors and ZnCl_2_ on the cleavage of the ED substrate. Line plots showing the degradation (*F*/min) of the ED substrate before and after the addition of EDTA (A), Pefabloc (B), BAM (C), NEM (D), and ZnCl_2_ (E). The red and blue colors indicate degradation before and after addition of the chemical compounds, respectively. Paired analyses were performed using Wilcoxon signed-rank test. Significance levels are depicted with asterisks: *p < 0.05, **p < 0.01, and ***p < 0.001. Abbreviation: AA = advanced adenoma, BAM = benzamidine, C = controls, CRC = colorectal carcinoma, EDTA = ethylenediaminetetraacetic acid, nAA = nonadvanced adenoma, and NEM = *N*-ethylmaleimide.

Proteolytic cleavage of the l-aspartic acid–l-glutamic acid (ED) substrate was significantly decreased in fecal samples of both AA and CRC patients compared to the controls. To gain insight into its identity and to biochemically characterize the protease, we examined the effect of several protease inhibitors and Zn^2+^ ions on degradation of the ED substrate. The addition of EDTA, a metalloprotease inhibitor, had minimal effect on protease activity in AA and nAA patients and controls but significantly increased activity in feces of CRC patients (Figure 3A). In contrast, the serine protease inhibitor Pefabloc significantly decreased the level of degradation of ED in the feces of CRC patients, with no notable alterations observed in the other groups (Figure 3B). After the addition of the trypsin-like serine and threonine protease inhibitor BAM, an increase in proteolytic activity was seen for all groups excluding nAA patients (Figure 3C). Conversely, a significant decrease in the level of degradation of the ED substrate was observed in all groups in the presence of NEM, which acts as a cysteine protease inhibitor (Figure 3D). Furthermore, in the presence of ZnCl_2_, decreased degradation was observed for all groups except AA patients (Figure 3E).

## Discussion

In the present study, we designed and tested several different colorectal neoplasia-specific fluorogenic substrates based on current insights from literature reporting on differential fecal amino acid composition in patients with colorectal neoplasia.^15,21^ We found that the degradation of the ED substrate significantly decreased in fecal samples of both AA and CRC patients, suggesting its potential as an adjuvant noninvasive diagnostic biomarker for advanced colorectal neoplasia.

Correlations were observed between the degradation profiles of the substrates and the human fecal proteome. However, specific proteases could not be identified due to the inability to match the correlated proteases based on the sequence motifs of the substrates. In addition, we found multiple correlations for carboxypeptidases and aminopeptidases. However, carboxypeptidases, including glutamate carboxypeptidase II which mainly cleaves the C-terminal glutamate residues from peptides, cannot cleave the substrates used because all are C-terminally flanked with LysDbc.^20^ The same applies to aminopeptidases; aminopeptidase A is known to cleave the N-terminal glutamate and to a lesser extend aspartate, whereas aminopeptidase N preferably cleaves after alanine but may be most amino acids; however, due to the presence of the FITC label at the N-terminal, the substrates will not be cleaved.^20^

To gain further insight into the biochemical characteristics of the associated proteases, additional experiments were conducted by using protease inhibitors and ZnCl_2_. The significant decrease in activity observed after NEM addition suggests that the cleavage of the ED substrate may potentially be mediated by cysteine proteases such as caspases or cathepsins. A modest correlation was observed between cathepsin H and the ED substrate. Cathepsin H is a cysteine protease that primarily acts as an aminopeptidase and, to a lesser extent, as an endopeptidase, particularly cleaving after arginine.^19^ This makes it less likely that cathepsin H can explain the ED substrate activity. Residual activity remains after the addition of NEM, which may be attributed to NEM saturation at a concentration of 12.5 mM. As stool is a rich source of various proteases, another explanation could be that multiple proteases from different classes were responsible for ED substrate cleavage. However, the absence of a decrease in protease activity following the addition of EDTA and BAM, and only a decrease for Pefabloc in CRC patients, does not support this explanation. Furthermore, we observed a decreased degradation of the ED substrate in the presence of ZnCl_2_ addition. This observation may be attributed to zinc binding at the calcium ion’s site, consequently deactivating the activity of calcium-dependent proteases. Therefore, it could be hypothesized that the proteases responsible for ED degradation might be calcium-dependent. It is worth noting that after the addition of BAM, an increase in the level of ED degradation was observed. This may be explained by BAM-inhibiting proteases that cleave the protease responsible for ED substrate activity.

Another six out of the 18 designed fluorogenic substrates tested within this study showed significantly decreased degradation in CRC patients. These substrates were correlated to fecal protease concentrations, as measured with LC–MS/MS in order to identify underlying proteases. Nevertheless, no specific protease could be identified based on correlation and known substrate preferences from the MEROPS and Uniprot database. A negative correlation was observed between TT substrate degradation and protease inhibitor A2ML1, which might suggest that A2ML1 inhibits the protease responsible for TT degradation. However, since A2ML1 can inhibit all 4 protease families, this does not provide insight into the biochemical characteristics of the proteases responsible for TT degradation.^24^ In addition, a combination of a negative correlation with trypsin inhibitor bikunin and a positive correlation with trypsin 2 was observed. However, trypsin 2 is an endopeptidase exhibiting selective cleavage preference after an arginine or lysine residue, and therefore it is unlikely that the TT degradation is attributed to trypsin 2.^19^

Recently, fecal proteolytic activity and the diagnostic potential of fluorogenic substrates have been described in pediatric patients with inflammatory bowel disease;^25^ however, literature describing this technique in fecal samples of patients with colorectal neoplasia is limited. Until now, protease activity profiling in colorectal neoplasia has predominantly been performed in tissue. To the best of our knowledge, our research is the first to introduce the novel approach of profiling protease activity in fecal samples, providing a more accessible and noninvasive matrix compared to tissue samples. In tissue, extracellular protease activity is primarily linked to tumorigeneses, as they are often overexpressed in malignant tissue.^13,14^ These kinds of observations led to the development of protease inhibitors as an anticancer therapeutics. However, no effect was seen in clinical trials investigating the efficacy of some protease inhibitors, and in some patients, there was even a suggestion of accelerated tumor growth.^26,27^ This leads to the hypothesis that some proteases may have antitumor properties, which aligns with our observations of a reduction in protease activity in the feces of patients with colorectal neoplasia compared to controls. For example, intracellular caspases, cysteine-type endopeptidases that mainly cleave after an aspartic acid residue, are shown to be involved in apoptosis. The caspase 8 gene (CASP8) is previously demonstrated to be inactivated in colorectal carcinomas and therefore might lead to the loss of apoptotic function and contribute to the pathogenesis of CRC.^28^ However, these mutations were not seen in low- and high-grade dysplastic colorectal adenomas. In addition, the literature is not consistent, as there are also studies describing upregulation of CASP8 in CRC tissue.^29,30^ This could be explained by its diverse role of influencing multiple cellular signaling pathways. Thus, CASP8, depending on the specific cellular context, may function as either a tumor suppressor or promoter.^31,32^ Furthermore, most of the cathepsin subtypes, also belonging to the family of cysteine proteases, are mainly found to be upregulated in colorectal cancer and associated with poor survival. Therefore, the hypothesis that cathepsins are involved in the substrate degradation is not obvious.^33^ However, given the diversity of subtypes within the cathepsin proteases, there is a possibility that a cathepsin with tumor-suppressive ability has simply not yet been identified and described but may be present and could account for our observations.

The vast majority of correlated human proteases measured by LC–MS/MS could not be assigned to substrate degradation; an explanation might be that LC–MS/MS measures protease abundance independent of whether they are active or inactive, whereas the FRET assay only measures biologically active proteases. Consequently, there may not be a direct correlation between the abundance and activity. The decreased activity in fecal samples of CRC patients could therefore, independent of protease abundance, be dependent on the micro environmental conditions which affect the activity of proteases, including local pH, and the presence of cofactors and inhibitors.^34^

This study has several limitations that need to be addressed. First, we were not able to include the bacterial proteome for protease identification. Stool is a rich source of microbes and microbe-derived proteins. Several studies have linked bacterial proteases to gastro-intestinal diseases, referring to the effect of a dysregulated balance between bacterial proteases, their host, and protease inhibitors to pathophysiological conditions.^35^ Therefore, it would be of added value to include the bacterial proteome in correlation with colorectal neoplasia-specific substrates in future research. Lastly, because of the relatively small sample size, the substrate activity as a diagnostic tool for CRC needs to be externally validated in a larger additional data set. Nonetheless, it is important to note that this was an exploratory study, and follow up studies that will address the above-mentioned limitations should be designed.

## Conclusions

In this study, colorectal neoplasia-specific fluorogenic substrates were successfully designed, highlighting ED as a potential noninvasive biomarker for detecting both AA and CRC patients. Although the responsible proteases for ED substrate cleavage in feces of AA and CRC patients remain to be identified, we hypothesize an association with calcium-dependent cysteine proteases. Further experiments are warranted for precise identification and validation of its diagnostic potential. Substrate activity measurement is relatively simple with high throughput, and therefore, once validated, these fluorogenic substrates hold promise as novel, adjuvant noninvasive diagnostic biomarker test for colorectal neoplasia.

## Data Availability

After completion and publication of the trial, coded data will be available from the corresponding author on reasonable request.

## Supporting Information Available

Comprehensive data including analytical HPLC chromatograms, MALDI mass spectrometry data, and overviews of protease correlations, substrate degradation comparisons, and effects of protease inhibitors and ZnCl_2_ (PDF)
