# Supplementary material for: Detecting Colorectal Neoplasia Using Specific Fecal Fluorogenic Protease-Sensitive Substrates: A Pilot Study
Source: Anal Chem. 2024 Dec 12;96(51):20239–46. doi: 10.1021/acs.analchem.4c04586 (PMC11672227; doi:10.1021/acs.analchem.4c04586)
Supplement: Supplementary file 1 — ac4c04586_si_001.pdf [file ac4c04586_si_001.pdf]

Supporting Information

Detecting colorectal neoplasia using specific fecal fluorogenic protease sensitive substrates: a pilot-study

Roza C.M. Opperman[1,2,3*], Sofie Bosch[1,2], Kamran Nazmi[4], Floris J. Bikker[4], Henk S. Brand[4], Connie R. Jimenez[3,5], Tim G.J. de Meij[2,6,7], Evelien Dekker[2,3,8], Nanne K.H. de Boer[1,2], Wendy E. Kaman[4]

[1]Department of Gastroenterology and Hepatology, Amsterdam UMC, Vrije Universiteit Amsterdam, 1081 HV Amsterdam, The Netherlands.

[2]Amsterdam Gastroenterology Endocrinology Metabolism (AGEM) Research Institute, 1081 HV Amsterdam, The Netherlands.

[3]Cancer Center Amsterdam, research program, 1081 HV Amsterdam, The Netherlands.

[4]Department of Oral Biochemistry, Academic Centre for Dentistry Amsterdam, University of Amsterdam and VU University Amsterdam, Gustav Mahlerlaan 3004, 1081 LA Amsterdam, The Netherlands.

[5]Department of Medical Oncology, Amsterdam UMC, VU University Medical Center, 1081 HV Amsterdam, The Netherlands.

[6]Department of Pediatric Gastroenterology, Emma Children's Hospital, Amsterdam UMC, Vrije Universiteit Amsterdam, 1081 HV Amsterdam, The Netherlands.

[7]Department of Pediatric Gastroenterology, Emma Children's Hospital, Amsterdam UMC, Academic Medical Centre, 1105 AZ Amsterdam, The Netherlands.

[8]Department of Gastroenterology and Hepatology, Amsterdam UMC, University of Amsterdam, 1081 HV Amsterdam, the Netherlands.

Table of contents

Topic	Page
**Supporting Figure 1.** Analytical HPLC chromatograms of the FRET-peptide substrates synthesized in this study.	S2
**Supporting Figure 2.** Maldi data of the FRET-peptide substrates synthesized in this study.	S3
**Supporting Table 1**. Overview of significant correlated proteases and protease inhibitors with substrate degradation	S4
**Supporting Table 2**. Overview of substrate degradation for the different comparisons with corresponding median and p-values	S6
**Supporting Table 3**. Overview substrate degradation before and after addition of protease inhibitors and ZnCl2	S7

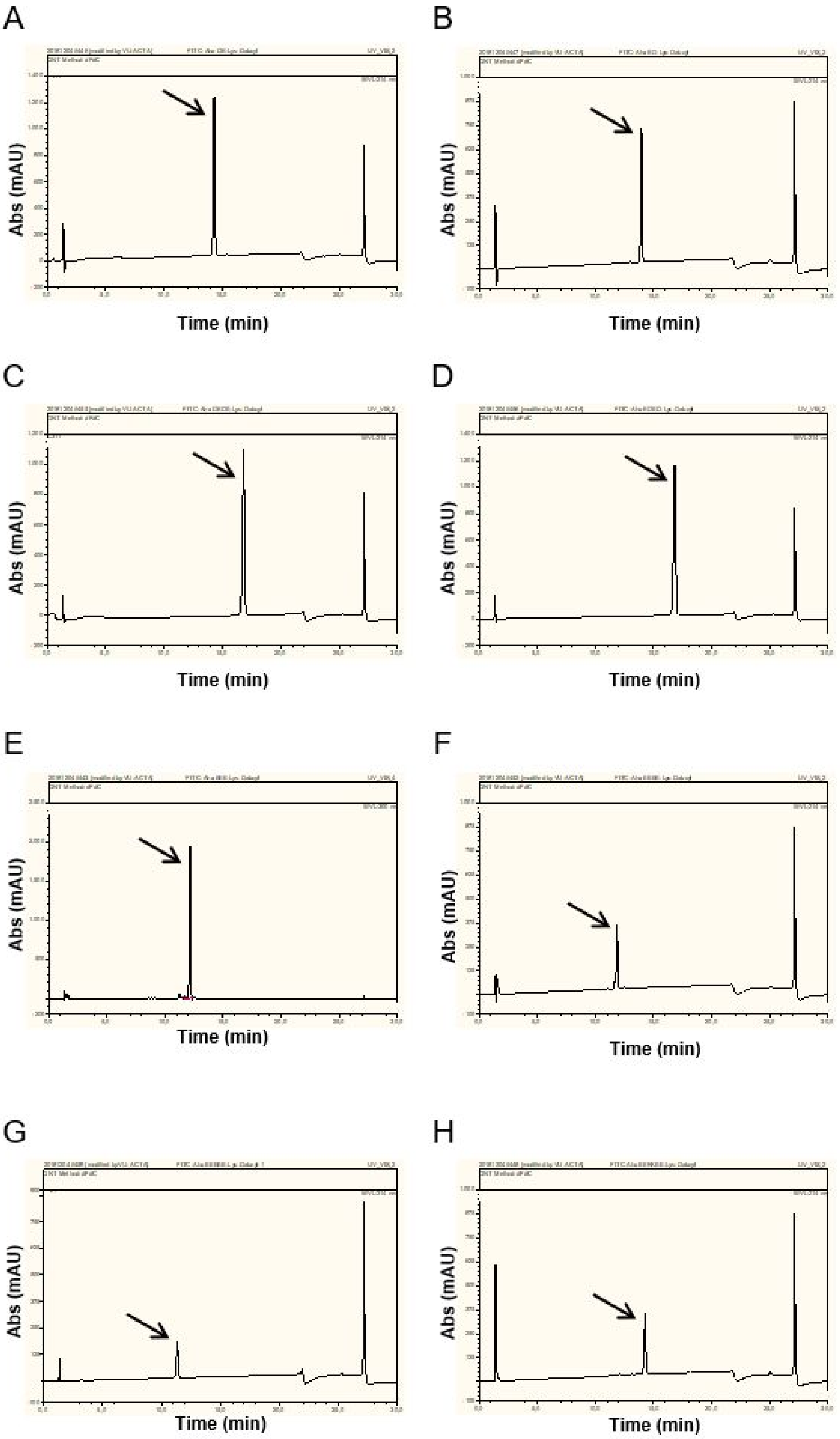

Supporting Figure 2. Analytical HPLC chromatograms of the FRET-peptide substrates synthesized in this study.
Absorbance was measured at 214 nm. Substrates analysed are DE (A), ED (B), DEDE (C), EDED (D), EEE (E), EEEE (F), EEEEE (G) and EEKKEE (H). Results confirm purity of the FRET-peptide substrates. Early and late peaks are caused by pressure differences due to starting and ending runs in the HPLC.

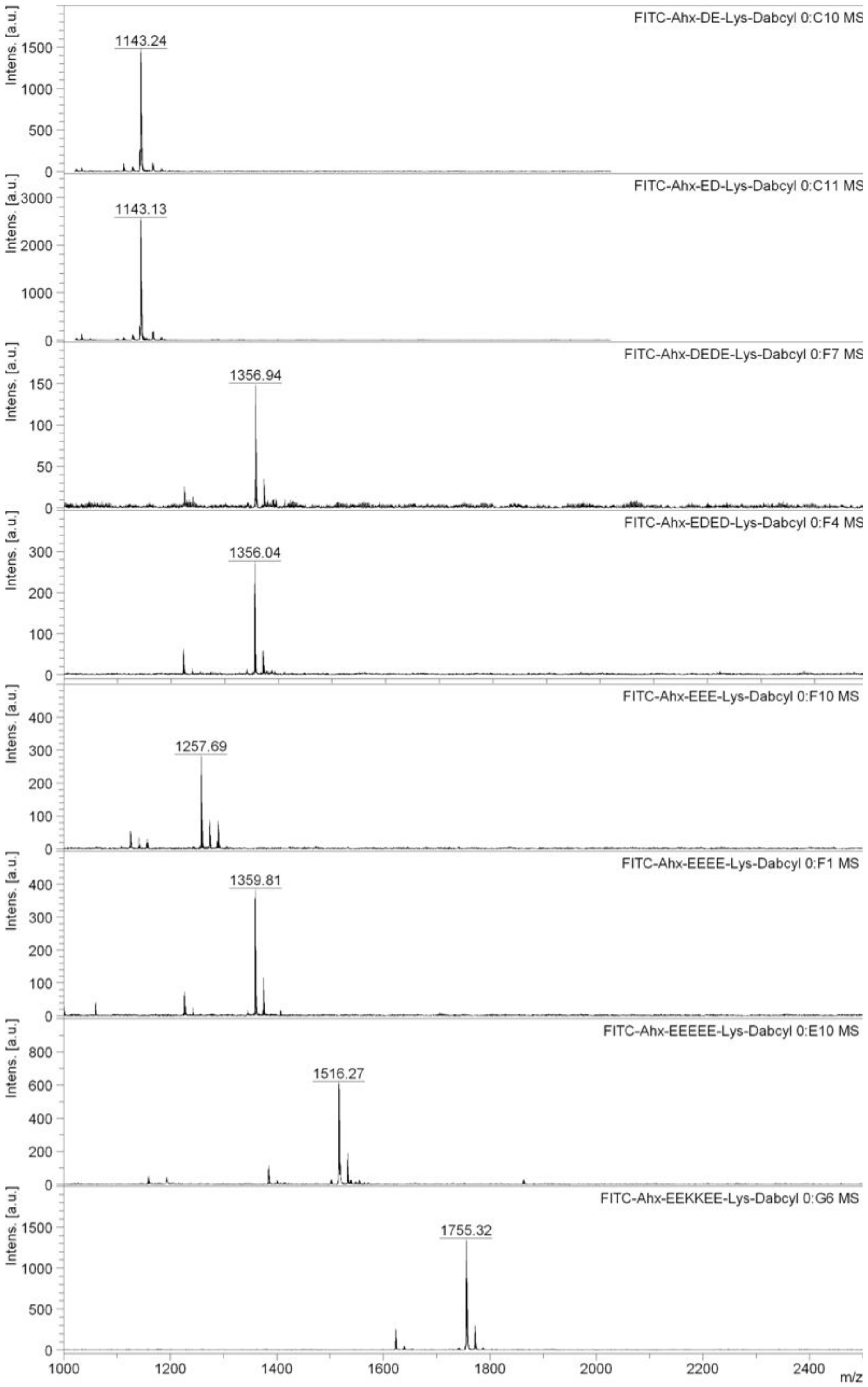

Supporting Figure 2. Maldi mass spectrometry data of the FRET-peptide substrates synthesized in this study.

Results show that the molecular weights of the FRET- peptide substrates correspond with the calculated molecular weights (mass in kDa).

Supporting Table 1. Overview of significant correlated proteases and protease inhibitors with substrate degradation [a].

Proteases	Statisitcs	ED	EEE	EEEE	EEEEE	DD	TT	HH
Aminopeptidase A	Spearman Correlation	0.011	0.219	0.401	0.47	0.084	-0.069	0.115
	Sig, (2-tailed)	0.942	0.149	0.006	0.001	0.583	0.653	0.452
Aminopeptidase N	Spearman Correlation	0.421	0.379	0.389	0.375	0.255	0.308	0.473
	Sig, (2-tailed)	0.004	0.01	0.008	0.011	0.091	0.04	0.001
Carboxypeptidase B	Spearman Correlation	0.442	0.315	0.417	0.379	0.162	0.307	0.325
	Sig, (2-tailed)	0.003	0.036	0.005	0.011	0.287	0.04	0.03
Carboxypeptidase O	Spearman Correlation	-0.046	0.225	0.257	0.34	0.151	-0.067	-0.012
	Sig, (2-tailed)	0.762	0.138	0.088	0.022	0.321	0.66	0.94
Cathepsin H	Spearman Correlation	0.316	0.435	0.428	0.432	0.382	0.332	0.308
	Sig, (2-tailed)	0.035	0.003	0.003	0.003	0.01	0.026	0.04
Cathepsin S	Spearman Correlation	0.268	0.38	0.295	0.284	0.382	0.202	0.297
	Sig, (2-tailed)	0.075	0.01	0.049	0.058	0.01	0.184	0.048
Chymotrypsin-C	Spearman Correlation	-0.099	-0.333	-0.182	-0.114	-0.353	-0.165	-0.264
	Sig, (2-tailed)	0.516	0.026	0.231	0.454	0.018	0.277	0.08
Cytosol alanyl aminopeptidase	Spearman Correlation	-0.34	-0.146	-0.073	-0.089	-0.182	-0.234	-0.094
	Sig, (2-tailed)	0.022	0.337	0.633	0.561	0.233	0.123	0.539
Dipeptidyl peptidase IV	Spearman Correlation	0.12	0.217	0.223	0.308	0.017	0.001	0.09
	Sig, (2-tailed)	0.432	0.151	0.141	0.04	0.913	0.996	0.555
Elastase II	Spearman Correlation	-0.195	-0.097	-0.081	-0.067	-0.156	-0.376	-0.347
	Sig, (2-tailed)	0.2	0.527	0.597	0.661	0.306	0.011	0.019
Enteropeptidase	Spearman Correlation	0.331	0.44	0.529	0.52	0.32	0.26	0.432
	Sig, (2-tailed)	0.026	0.002	0	0	0.032	0.085	0.003
Glutamate carboxypeptidase II	Spearman Correlation	0.178	0.459	0.422	0.466	0.211	0.016	0.225
	Sig, (2-tailed)	0.241	0.002	0.004	0.001	0.164	0.917	0.137
Pancreatic elastase II	Spearman Correlation	0.219	0.257	0.15	0.122	0.201	0.447	0.482
	Sig, (2-tailed)	0.147	0.089	0.325	0.423	0.184	0.002	0.001
Pancreatic endopeptidase E	Spearman Correlation	-0.12	-0.301	-0.242	-0.176	-0.302	-0.093	-0.263
	Sig, (2-tailed)	0.433	0.045	0.109	0.247	0.044	0.542	0.081
Pancreatic endopeptidase E form B	Spearman Correlation	0.284	0.135	0.158	0.183	0.028	0.407	0.408
	Sig, (2-tailed)	0.058	0.378	0.3	0.23	0.853	0.006	0.005
Prolyl oligopeptidase	Spearman Correlation	-0.34	-0.146	-0.073	-0.089	-0.182	-0.234	-0.094
	Sig, (2-tailed)	0.022	0.337	0.633	0.561	0.233	0.123	0.539
Trypsin 2	Spearman Correlation	0.326	0.1	0.064	0.022	0.269	0.404	0.285
	Sig, (2-tailed)	0.029	0.513	0.675	0.884	0.074	0.006	0.058
**Protease inhibitors**	**Statisitcs**	**ED**	**EEE**	**EEEE**	**EEEEE**	**DD**	**TT**	**HH**
Alpha-2-macroglobulin-like protein 1	Spearman Correlation	-0.277	-0.131	0.032	-0.023	-0.224	-0.347	-0.16
	Sig, (2-tailed)	0.066	0.389	0.832	0.881	0.138	0.019	0.293
Antithrombin	Spearman Correlation	0.175	0.504	0.348	0.362	0.352	0.067	0.056
	Sig, (2-tailed)	0.249	0	0.019	0.015	0.018	0.661	0.713

Bikunin	Spearman Correlation	-0.168	0.023	-0.03	-0.058	-0.058	-0.295	-0.21
	Sig, (2-tailed)	0.269	0.878	0.844	0.707	0.707	0.049	0.166
Cystatin A	Spearman Correlation	0.282	0.298	0.226	0.267	0.01	0.203	0.351
	Sig, (2-tailed)	0.061	0.047	0.136	0.077	0.947	0.18	0.018
Serpin B1	Spearman Correlation	0.169	0.3	0.31	0.277	0.268	-0.006	0.171
	Sig, (2-tailed)	0.268	0.045	0.038	0.066	0.075	0.971	0.262
Serpin B3	Spearman Correlation	0.216	0.301	0.287	0.242	0.237	0.092	0.284
	Sig, (2-tailed)	0.154	0.045	0.056	0.109	0.118	0.548	0.059
Serpin B10	Spearman Correlation	0.219	0.295	0.152	0.084	0.096	0.172	0.175
	Sig, (2-tailed)	0.148	0.049	0.319	0.585	0.533	0.26	0.252

[a] Summary of Spearman correlation analysis showing the correlation coefficient and p-value of the correlation between the concentration of proteases and protease inhibitors, measured with LC-MS/MS, and substrate degradation, measured with a FRET assay. Significant p-values are highlighted in green, with darker shades indicating a more significant correlation.

Supporting Table 2. Overview of substrate degradation for the different comparisons with corresponding median and p-values [a].

Substrate	Control<br>Median [IQR]	nAA<br>Median [IQR]	AA<br>Median [IQR]	CRC<br>Median [IQR]	Control vs nAA<br>p_value	AA vs nAA<br>p_value	CRC vs nAA<br>p_value	AA vs Control<br>p_value	CRC vs Control<br>p_value	CRC vs AA<br>p_value
**DD**	23.9 [22.1]	21.9 [19.9]	27.3 [16.2]	13.6 [9.3]	0.666	0.549	0.497	0.975	0.046	0.082
**Dd**	4.5 [3.4]	6 [8.3]	6.5 [6.2]	5.8 [7]	0.403	0.72	0.51	0.781	0.781	0.651
**EE**	8 [7.6]	6.5 [11.3]	4.1 [6.6]	4.7 [6]	0.977	0.968	0.575	0.516	0.368	0.804
**Ed**	2.1 [3.5]	2.2 [4.2]	5.4 [5.3]	2.1 [5.3]	0.837	0.78	0.843	0.329	0.938	0.413
**ED**	373.4 [264.1]	194.8 [132.5]	184.8 [210.2]	126.4 [115.8]	0.154	0.447	0.283	0.023	0.027	1
**DE**	127.2 [69]	103.4 [77.3]	85.1 [59]	59.2 [97.1]	0.585	0.356	0.123	0.159	0.085	0.422
**PP**	43.2 [44.5]	47.9 [36.6]	38.5 [75.7]	44.1 [33]	0.437	0.604	0.456	0.734	0.82	0.917
**SS**	41 [39.9]	39.1 [32.0]	34.7 [26]	30.7 [38.5]	0.709	0.968	0.582	0.643	0.212	0.508
**VV**	54.8 [18.4]	42.3 [18.0]	29.8 [51.5]	28.2 [27.9]	0.096	0.661	0.283	0.277	0.06	0.808
**TT**	92.7 [52]	46.6 [39.6]	55 [40.2]	44.2 [40.3]	0.122	0.968	0.582	0.062	0.009	0.464
**HH**	31.6 [107.9]	9.6 [12.7]	7.6 [21.8]	5.7 [6.6]	0.154	0.78	0.228	0.062	0.004	0.422
**Hd**	3.3 [6.5]	4.0 [10.6]	7.4 [8.5]	4.8 [8]	0.709	0.72	0.717	0.403	0.979	0.455
**EEE**	201.3 [155.6]	79.3 [105.1]	175.1 [201.3]	66.2 [77.3]	0.016	0.156	0.771	0.6	0.009	0.096
**EEEE**	1330.3 [730.7]	841.3 [887.2]	1229.8 [123.3]	660.8 [566.4]	0.108	0.78	0.418	0.439	0.004	0.096
**EEEEE**	1196.1 [427.5]	869.8 [927]	1185.1 [247.0]	631 [698.9]	0.259	0.968	0.346	0.439	0.005	0.096
**EDED**	1171.5 [1239.7]	1283.3 [1097.5]	1174 [456.7]	585 [537.7]	0.886	0.549	0.14	0.643	0.095	0.277
**DEDE**	1286.5 [1097.6]	1508.3 [649.8]	1474.1 [344.4]	1227.4 [1046.6]	0.371	0.78	0.283	0.688	0.899	0.602
**EEKKEE**	127.2 [89.5]	93.3 [117.4]	104 [98.2]	92.8 [111.1]	0.472	1	0.974	0.477	0.297	0.972

[a] Overview of median substrate degradation rates (f/min) and results of unpaired univariate analyses using the Mann-Whitney U test. Abbreviations: AA = advanced adenoma, CRC = colorectal cancer, IQR = inter quartile range and nAA = non-advanced adenoma.

Supporting Table 3. Overview substrate degradation before and after addition of protease inhibitors and ZnCl2 [a].

	Control			nAA			AA			CRC		
	Control median [IQR]	Compound median [IQR]	p_value	Control median [IQR]	Compound median [IQR]	p_value	Control median [IQR]	Compound median [IQR]	p_value	Control median [IQR]	Compound median [IQR]	p_value
**EDTA**	178.9 [208.8]	170.4 [218.8]	0.808	119.7 [68.5]	119.5 [80.6]	0.492	132 [87.1]	133.7 [98.3]	0.164	95.6 [80.7]	110.2 [90.4]	0.027
**BAM**	165.5 [207.6]	197.2 [308.4]	<0.001	99.4 [115.9]	142.5 [96.2]	0.275	123.8 [75.9]	188.2 [105.8]	0.004	107 [87.6]	140.3 [82.7]	<0.001
**Pefabloc**	165.5 [207.6]	154 [132]	0.241	99.4 [115.0]	120.2 [105.6]	0.695	123.8 [75.9]	142.1 [60.7]	0.055	107 [87.6]	83.1 [51.7]	0.016
**NEM**	165.5 [207.6]	118.2 [147.1]	0.001	99.4 [115.9]	72.2 [37.3]	0.002	123.8 [75.9]	92.3 [44.6]	0.004	107 [87.6]	75.6 [44.4]	<0.001
**ZnCl2**	165.5 [207.6]	121.2 [225.5]	0.017	99.4 [115.9]	82.8 [96.7]	0.002	123.8 [75.9]	85.4 [132.8]	0.164	107 [87.6]	84.5 [83.5]	<0.001

[a] Overview of the differences in proteolytic degradation (f/min) before and after addition of protease inhibitor analysed using the Wilcoxon signed-rank test. A p-value ≤ 0.05 was considered significant. Abbreviations: AA = advanced adenoma, BAM = benzamidine, CRC = colorectal cancer, EDTA = ethylenediaminetetraacetic acid, IQR = inter quartile range, nAA = non-advanced adenoma and NEM = N-Ethylmaleimide.
